# Automated Classification of Resting-State fMRI ICA Components Using a Deep Siamese Network

**DOI:** 10.3389/fnins.2022.768634

**Published:** 2022-03-18

**Authors:** Yiyu Chou, Catie Chang, Samuel W. Remedios, John A. Butman, Leighton Chan, Dzung L. Pham

**Affiliations:** ^1^Center for Neuroscience and Regenerative Medicine, Bethesda, MD, United States; ^2^Department of Electrical Engineering and Computer Science, Vanderbilt University, Nashville, TN, United States; ^3^Department of Computer Science, Johns Hopkins University, Baltimore, MD, United States; ^4^Radiology and Imaging Sciences, National Institutes of Health, Bethesda, MD, United States; ^5^Rehabilitation Medicine Department at Clinical Center, National Institutes of Health, Bethesda, MD, United States

**Keywords:** resting-state functional MRI, independent component analysis, deep learning, siamese network, classification, magnetic resonance imaging (MRI), one-shot learning

## Abstract

Manual classification of functional resting state networks (RSNs) derived from Independent Component Analysis (ICA) decomposition can be labor intensive and requires expertise, particularly in large multi-subject analyses. Hence, a fully automatic algorithm that can reliably classify these RSNs is desirable. In this paper, we present a deep learning approach based on a Siamese Network to learn a discriminative feature representation for single-subject ICA component classification. Advantages of this supervised framework are that it requires relatively few training data examples and it does not require the number of ICA components to be specified. In addition, our approach permits one-shot learning, which allows generalization to new classes not seen in the training set with only one example of each new class. The proposed method is shown to out-perform traditional convolutional neural network (CNN) and template matching methods in identifying eleven subject-specific RSNs, achieving 100% accuracy on a holdout data set and over 99% accuracy on an outside data set. We also demonstrate that the method is robust to scan-rescan variation. Finally, we show that the functional connectivity of default mode and salience networks identified by the proposed technique is altered in a group analysis of mild traumatic brain injury (TBI), severe TBI, and healthy subjects.

## Introduction

Examining the human brain as an integrative network of functionally interacting brain regions can provide new insights about large-scale neuronal communication in the human brain. It also provides a platform to examine how functional connectivity and information integration relates to human behavior and how this organization may be altered in neurodegenerative diseases. Independent component analysis (ICA) is a powerful mathematical tool for simultaneously extracting a variety of coherent functional networks (Beckmann and Smith, 2004; Calhoun and Adali, 2006), while separating them from different sources of noise induced by head motion or physiological confounds (Salimi-Khorshidi et al., 2014). It decomposes resting-state functional magnetic resonance imaging (rsfMRI) data into distinct networks that are temporally correlated but maximally independent in the spatial domain. ICA has been successfully used to study a wide variety of neurological conditions such as Alzheimer’s disease (Dennis and Thompson, 2014), multiple sclerosis (Castellazzi et al., 2018), and traumatic brain injury (Iraji et al., 2015; Li et al., 2020).

There are two significant challenges in the application of ICA for functional connectivity analysis. First, the output of ICA decomposition depends on a fundamental parameter: the total number of independent components. The optimal choice of this parameter, related to determining the effective data dimension, remains an open question. Choosing too small a value for the effective data dimension might under-decompose the signal and generate “fused components.” At the same time, choosing too high a value might over-decompose the data and lead to splitting of meaningful components (Li et al., 2007; Hui et al., 2013). The other major challenge is that ICA does not provide any labeling or ordering of its components, and the user must therefore determine how the functional network corresponds to each component. When dealing with large data sets, this manual process can be labor intensive and requires expertise. One possible way to deal with these challenges is by running ICA with different numbers of components and selecting the one that extracts the best-fitted result for each desired functional network (Kairov et al., 2017). Therefore, a fully automatic algorithm that can reliably classify ICA components into various types of functional brain networks while also generating a goodness-of-fit or confidence metric would provide a unified solution to these two challenges.

One approach to mitigate the burden of identifying resting state networks (RSNs) in large data sets is to focus on group analyses. However, there is an increasing need for subject-specific analyses, such as for surgical planning (Catalino et al., 2020), brain stimulation (Jung et al., 2020), and in studies where group bias may be a concern (Michael et al., 2014). For these situations (or analyses), one can apply an automated approach for identifying particular networks from subject-specific ICA decompositions. Template matching has been the most commonly used method for automatic ICA identification in the last two decades. In (Demertzi et al., 2014), a Pearson correlation was computed between the template and each ICA component, and the network corresponding to the template yielding the highest correlation was selected as the identified network. In (Greicius et al., 2004, 2007), a linear template-matching procedure was applied, which involves taking the average z-score of voxels falling within the template minus the average z-score of voxels outside the template and selecting the component in which this difference (the goodness-of-fit) was the greatest.

Deep learning using Convolutional Neural Networks (CNNs) has become the state-of-the-art for image segmentation, classification, detection and retrieval related tasks (Khan et al., 2020). Recent work (Zhao et al., 2018) has demonstrated the capability of CNNs for automatic recognition of spatial resting-state network maps, although this and other machine learning approaches for subject-specific identification of RSNs (Hacker et al., 2013; Lv et al., 2015) were not designed to work with ICA. In Vergun et al., 2016, several machine learning approaches, including a one layer neural network, were used to label ICA components in epilepsy, achieving up to 90% accuracy. In Nozais et al., 2021, a multilayer perceptron deep neural network approach for identifying 45 RSNs based on ICA was implemented using two databases of over 250 subjects for training and over 1,900 subjects for testing, achieving an accuracy of 92%. In our previous study (Chou et al., 2018), we proposed a fully automatic deep 3D 2-channel CNN method based on stacks of inception modules (denoted as inception CNN) that can reliably identify ICA components corresponding to eight major functional networks with 98% accuracy rate over 108 testing samples.

Convolutional Neural Networks can be prone to erroneously giving high-confidence predictions to out-of-distribution examples– i.e., inputs that are markedly different from any samples in the data used to train the CNN, and that do not correspond to any particular class the CNN was trained to predict (Wang et al., 2017). A seemingly straightforward approach to handle this issue is to enlarge the training set of both in- and out-of-distribution examples. However, the number of out-of-distribution examples can be infinitely many, making training computationally expensive and intractable, since its underlying space is unbounded. Moreover, ensuring accurate classification of in-distribution samples and correct detection out-of-distribution samples may require exceedingly large architectures, further complicating the training process (Liang et al., 2018). To handle these issues, we (Chou et al., 2019) explored a semi-supervised Generative Adversarial Network (denoted as sGAN) (Augustus, 2016) for the classification on ICA components by using a shared discriminator/multi-class classifier that discriminates real data from synthetic while also predicting the class label. The sGAN achieved a high accuracy rate (98.7%) for eight functional networks without requiring additional labeled images during training or access to out-of-distribution examples. The trained generator could also be used to synthesize realistic functional networks as a source of data augmentation. However, obtaining a large enough training set for the sGAN was challenging, requiring 501 ICA manually labeled components. To extend the sGAN to classify RSNs beyond the 8 components would require a substantial number of additional components to be labeled for training.

Poor performance on a limited labeled data set is a common problem with CNNs (O’Mahony et al., 2019). In medical imaging applications, collecting this much data may not always be feasible. Furthermore, there are situations in which a user is interested in an additional (new) classification label outside the set of original labels. Standard approaches require that the network be completely retrained in order to add a new label. To circumvent these limitations, we propose to use a metric learning approach based on a deep Siamese Network (called SiameseICA) for the classification of ICA components with a relatively small amount of training data. Siamese networks provide a framework to learn metric embeddings that provide a discriminative distance function between classes. The yielded distance metric is able to enlarge the separability of samples from different classes and reduce the variations of samples from the same class simultaneously. By learning distance functions rather than explicitly learning classification, metric learning approaches have successfully been used for classification yielding state-of-the-art performance on problems with small numbers of labeled samples without overfitting (Hoffer and Ailon, 2014; Cao et al., 2020). Siamese Networks have been applied to various problems, including image recognition and verification, visual tracking, novelty and anomaly detection, one-shot and few-shot learning (Bromley et al., 1994; Koch et al., 2015; Schroff et al., 2015; Bertinetto et al., 2016; Snell et al., 2017; Rana and Kisku, 2020). They are useful in cases where there are large numbers of classes with small numbers of observations of each because they avoid the problem of directly classifying an image. Rather, they take two images as inputs and compute feature vectors in a low-dimensional space to measure the similarity between the images. We show how this can be used to address the issues of labeling ICA components and determining the number of components. Although SiameseICA can be applied for group ICA analysis, we focus our evaluation primarily on single subject ICA because this leads to noisier components that are typically more difficult to identify.

This paper makes the following contributions: (1) we propose a novel technique for ICA classification with low training samples that outperformed CNN based deep learning methods and traditional template matching methods; (2) we demonstrate that the model can generalize to new categories, unseen in the training process; (3) we show that the best-fit ICA components representing the functional networks can be effectively identified by the proposed technique, thereby removing the requirement to know the number of ICA components *a priori*; (4) we show that our technique is robust to the scan-rescan variation, demonstrating high reproducibility on classifying ICA components; (5) we perform an analysis on mild TBI, severe TBI and healthy subjects using networks identified by the proposed technique and show significant group differences. The source code of SiameseICA, as well as a trained model, is available on Github: http://github.com/chouyiyu/deepICAclassifier.

## Materials

Four sets of data were used to train and evaluate the proposed method. The first dataset, denoted as CNRM, consisted of 179 scans including 41 healthy, 93 mild-TBI and 45 severe-TBI subjects enrolled in a natural history study of TBI and scanned on a Siemens Biograph mMR 3T scanner. The rsfMRI images (TR = 2,000 ms, TE = 27 ms, flip angle = 90 degrees, voxel size = 3.43 mm × 3.43 mm × 3 mm, dimensions = 64 × 64 × 36, Time points = 206) were used for training, testing (sections “Classification Performance,” “One-Shot Classification for the Caudate Network”) and TBI group analysis (section “Group comparison between mild TBI, severe TBI and healthy subjects”). T1-weighted MPRAGE images (TR = 2,530 ms, TE = 3 ms, flip angle = 7 degrees, TI = 1,100 ms, voxel size = 1 mm × 1 mm × 1mm, dimensions = 256 × 256 × 176) were also used for registration purposes. Participants in the study were enrolled under a protocol approved by the institutional review board at the National Institutes of Health and consented to research use of their imaging data. Training data were used for optimizing the SiameseICA network parameters, and the test data were used to examine the accuracy of identifying a single ICA component. For training and testing data, 30 ICA components were extracted from each subject using MELODIC (Multivariate Exploratory Linear Optimized Decomposition into Independent Components) (Beckmann and Smith, 2004) and manually identified *via* visual inspection as being one of eleven resting-state networks (RSNs): default mode, medial visual, occipital visual, lateral visual, motor, cerebellum, auditory, executive, salience, left dorsal attention and right dorsal attention. RSNs were only selected when they were identified with high confidence. Since some components may represent split or merged networks, all 11 RSNs were not necessarily identified for each subject. In addition to individual subject ICA components, group ICA processing was performed by creating random subsets of *n* subjects to generate additional training and testing image sets with more reproducible and fully-formed network representations (Zuo et al., 2010). The final sizes of the training set and testing set were 20 and 50, respectively, for each of the eleven RSNs considered.

The second dataset consisted of rsfMRI from 10 healthy subjects acquired using a GE Signa 3T scanner (TR = 2,000 ms, voxel size = 3.75 mm × 3.75 mm × 4 mm, dimensions = 64 × 64 × 36, Time points = 175), denoted as Milwaukee-b, obtained from the 1,000 Functional Connectomes Project (FCP) at http://fcon_1000.projects.nitrc.org/fcpClassic/FcpTable.html, Biswal et al. (2010). A T_1_-weighted MPRAGE image (voxel size = 0.938 mm × 0.938 mm × 1 mm, dimensions = 256 × 256 × 144; detailed information not available) was also acquired for each subject. This dataset was used for demonstrating the generalizability and separability between classes of the proposed SiameseICA method (section “Generalizability, separability and reproducibility”).

The third dataset (section “Classification Performance”), denoted as BeijingFCP, was also obtained from FCP and includes 198 healthy subjects with 621 independent components manually labeled for the validation. MRI was acquired using a Siemens Trio 3T scanner. Specifically, an echo-planar imaging (TR = 2,000 ms, voxel size = 3.125 mm × 3.125 mm × 3.6 mm, dimensions = 64 × 64 × 33, Time points = 225) sequence was performed to obtain resting state fMRI images. A T_1_-weighted MPRAGE (voxel size = 1.0 mm × 1.0 mm × 1.0 mm, dimensions = 256 × 256 × 176; detailed information not available) was carried out to acquire a high-resolution anatomical image of the brain structure.

The fourth dataset, denoted as MMRR-21 (section “Group Comparison Between Mild TBI, Severe TBI and Healthy Subjects”) consists of scan-rescan image sessions performed on a Philips Achieva 3T (TR = 2,000 ms, TE = 30 ms, flip angle = 75 degrees, voxel size = 3 mm × 3 mm × 3.973 mm, dimensions = 80 × 80 × 37, Time points = 210) on 21 healthy volunteers^1^. This dataset was intended to be a resource for statisticians and imaging scientists to be able to quantify the reproducibility of their imaging methods. Each subject from this dataset also had a T_1_-weighted MPRAGE image (TR = 6.7 ms, TE = 3.1 ms, TI = 842 ms, flip angle = 8 degrees, voxel size = 1 mm × 1 mm × 1.2 mm, dimensions = 256 × 256 × 170).

## Methods

This section provides details on the proposed method for identifying RSNs from the output of an ICA algorithm. The method comprises four major stages: (1) pre-processing; (2) application of ICA; (3) training the SiameseICA with a triplet loss function; and (4) finding the most likely class for a test image using the trained model.

### Image Preprocessing

Functional and structural T_1_-weighted MRI data were pre-processed using AFNI (*Cox, 1996*). The first five volumes were discarded, and the remaining images underwent de-spiking, slice timing correction, and finally motion correction using volume registration. The rsfMRI images were then rigidly co-registered to the subject’s own T1-weighted MPRAGE image and non-linearly warped into a standard (MNI) anatomical space. Gaussian smoothing with an 8mm full-width-half-magnitude (FWHM) kernel was then performed to spatially smooth the rsfMRI data and the final resolution of all images was resampled to 4 mm × 4 mm × 4 mm.

### Independent Component Analysis Extraction of Networks

**FIGURE 1:**
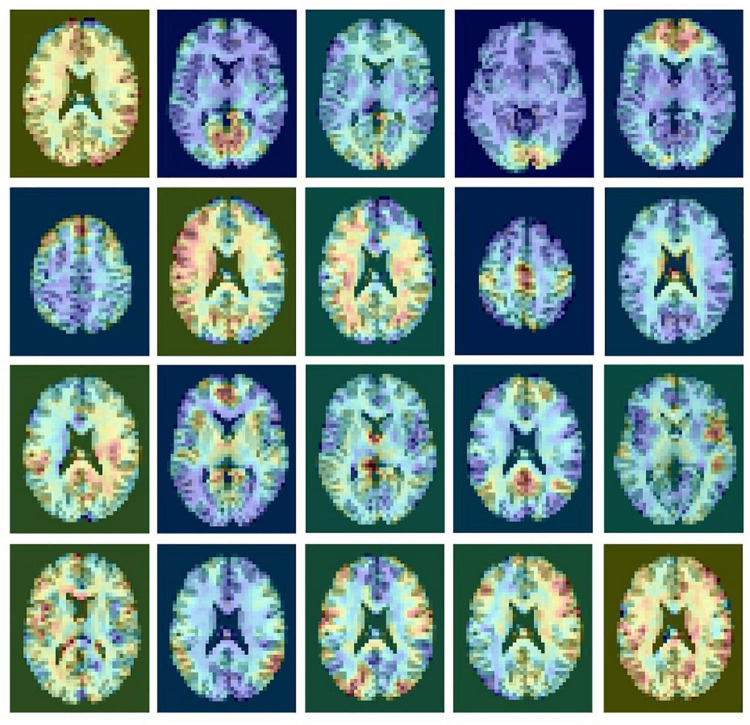
An example of a single subject ICA decomposition with 20 outputs showing rsfMRI signals and noise components. ICA does not provide any labeling of the components.

The pre-processed rsfMRI data were decomposed into multiple components using MELODIC from the FMRIB Software Library (FSL) to extract spatial maps of functional brain networks. MELODIC models the data as a linear mixture of spatiotemporal processes corrupted by noise and estimates maximally independent spatial sources that potentially represent functional networks. For each subject, multiple components were extracted and scaled to represent a spatial z-score map. In each spatial map, the z-score value associated with a given voxel reflects the weight of the independent component time course with respect to the relative measured BOLD data, thereby providing an indirect measure of functional connectivity. An example of a single subject ICA decomposition with 20 outputs is shown in Figure 1. Resting state networks in this output include the medial visual network (row 1, column 2); the occipital visual network (1.4); the executive network (2.1); the motor network (2.4); the default mode network (3.4); and the salience network (3.5).

### Siamese Network Architectures

Siamese Networks are a type of deep learning network composed of at least two parallel, identical Neural Networks (encoders). The parallel network architecture allows for the model to learn similarity, which can be used instead of a direct classification. This is the primary difference between the Siamese Network and a more traditional CNN architecture. At inference time, it takes two or more input images (or image sets) and computes a distance between those images. The computation of distance is learned from the training data by specifying which images are from the same RSN (small distance), and which images are from different RSNs (large distance). Each encoder that forms a part of the Siamese Network is designed to produce an embedding or a reduced dimensional representation of the input. These embeddings can then be used to optimize a ranking loss and, at inference time, used to compute distance. By comparing an unknown image against samples of labeled images, the classification is determined by the image with the smallest distance. This provides Siamese networks with the ability to learn classification tasks with low training samples, as well as to generalize to new, unseen data. In the following, the network structure of the encoder and triplet loss function are introduced for training Siamese Networks.

#### Encoder Network

**FIGURE 2:**
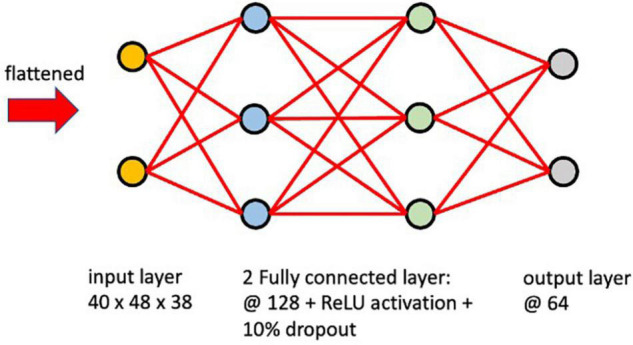
Network architecture of a single encoder.

The encoder network learns how to interpret the input image and compresses it into a vector as an embedded representation in a reduced dimensional space. These vectors encode the information and features that represent the input. In this study, the network architecture (Figure 2) of the encoder was defined with an input layer of the same shape as the input image (40 × 48 × 38), two fully connected layers with ReLU activation functions and dropout connections that randomly set input elements to zero with a 10% probability during training help prevent the network from overfitting (Srivastava et al., 2014). An output layer of 64 nodes produced our embedded representation. A learning rate of 0.0001 with the Adam optimizer was used and the model was trained with different epoch numbers and validated on a subset of the training set to determine that training for 500 epochs was sufficient. Since the location of the RSN activation is critical for classifying the ICA components, fully-connected layers were implemented instead of convolutional layers to avoid translation-invariance.

#### Triplet Loss Function

**FIGURE 3:**
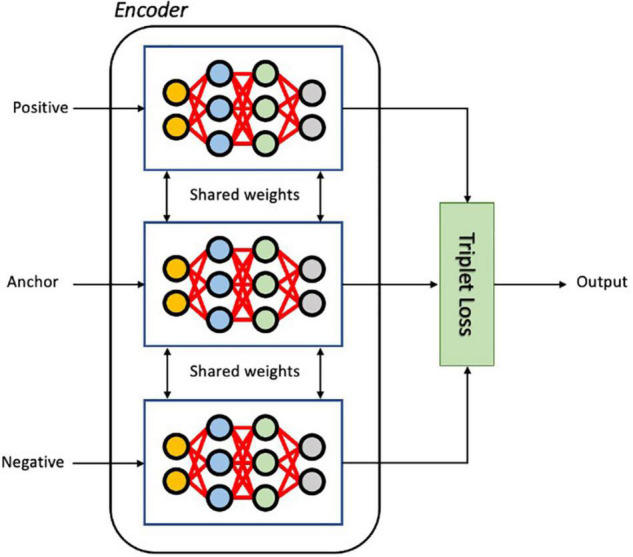
The architecture of the Siamese network with triplet loss function during training. As input, a triplet of images (Anchor, Positive and Negative) is given to three identical encoder networks. The embedded vectors of three input images are used to calculate the triplet loss.


The triplet loss aims to learn relative distances between samples, thereby defining an appropriate embedded representation for each class, a task often called metric learning (Hoffer and Ailon, 2014). In the embedding space, images from the same class should be close together and different classes should form well separated clusters. As illustrated in Figure 3, in each iteration of training, the input triplet (**a, p, n**) is sampled from the training set, where a baseline (anchor) input image volume a is compared to a positive input *p* (same class as the anchor) and a negative input *n* (a different class from the anchor). Then the triplet (**a**, **p**, **n**) is fed into the encoder network simultaneously to obtain the latent embeddings *f*(*a*),*f*(*p*)and*f*(*n*), where *f* defines a parametric function denoting the neural network encoder that maps high dimensional inputs (images *a*, *p*, and *n*) to a low-dimensional space. The loss for a triplet (**a, p, n**) can be formulated as (Wang et al., 2014):

L⁢(a,p,n)=max⁢{0,d⁢(a,p)-d⁢(a,n)+m},

Where *f*(*x*,*y*) = ||*f*(*x*)−*f*(*y*)||_2_ is the Euclidean distance between the latent vectors of image *x* and image *y*; *m* is the hyperparameter that controls the separation between similar and dissimilar vectors in the latent embedding. The triplet loss function encourages large distances between anchor and negative images while minimizing the distances between anchor and positive images, thereby learning to differentiate similar images from non-similar ones. With the triplet loss function, not only are inter-class feature differences enlarged, but also the intra-class features variations are reduced, allowing the discriminative power of the deeply learned features to be enhanced. In addition, these features are sufficiently generalized so as to distinguish new unseen classes. Training on triplets is beneficial since it produces more examples to train the model on, improving robustness to overfitting and the performance of the model.

### Using Siamese Network at Inference Time

**FIGURE 4:**
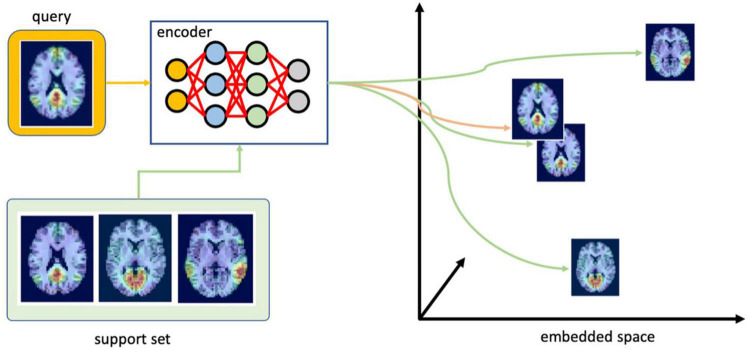
Finding the most likely class for the test image (query). First, the encoder embeds the query using the embedding function learned during training. Next, it compares this embedding with the support set to select the most likely class for the query (ICA components were overlaid with TT-N27 template for illustration purposes in this figure; the template is not incorporated into the data submitted to the CNN during training or inference).

As illustrated in Figure 4, at inference time, the input image (or query) of an unknown class is processed by the encoder to compute a vector in the latent space. This embedding is then compared with other vectors in the latent space representing different RSN clusters, known as the support set. This provides us with similarity scores or relative distances (equivalent by computing a multiplicative inverse) between the image with an unknown RSN and all of the existing clusters. To obtain a classification result, the RSN with the highest similarity (shortest distance) can be selected. If the input image does not belong to any of the clusters, the distances will be large between all image pairs.

Automated identification of the RSN can be used to surmount the challenge of determining the number of components when performing ICA to avoid split or merged RSNs. To identify the one ICA component that best represents each of the 11 desired RSNs, multiple runs of ICA with different numbers of components (from 20 to 99) were applied. The resulting components were then ordered based on the distances calculated by the proposed Siamese Network with the support set. The final neural network label was chosen as the one with the shortest distance for a specific support set.

## Results

### Classification Performance

SiameseICA was implemented on a Linux server using Keras (Chollet, 2016) with sixteen 32GB NVIDIA Tesla V100 graphics processing units and trained for 500 epochs with a batch size of 32. To stabilize the training process, the Adam optimizer was used with a learning rate 0.0001. The total training time was approximately 10 min with 20 training samples per ICA class and run time was 0.1 s. To demonstrate the effectiveness of the proposed model and parameter selection, different numbers of training samples (*N* = 2, 3, 4, 5, 10 and 20 for each class) were evaluated and compared with our previously proposed inception CNN (Chou et al., 2018) and sGAN (Chou et al., 2019) methods. Here the support set used at inference time to obtain the final classification results was selected from the group ICA training data of each RSN.

**FIGURE 5:**
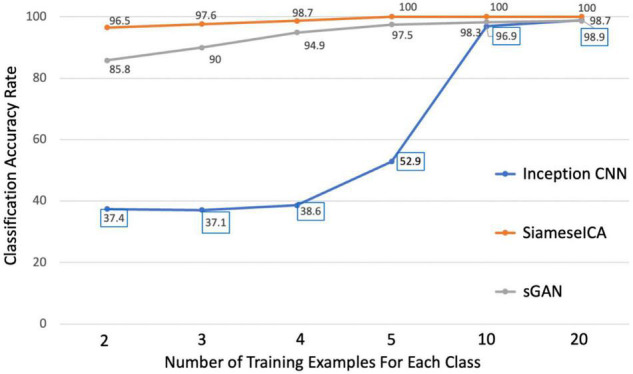
Plots of classification accuracy with different number of training examples for the inception CNN, sGAN and the proposed SiameseICA models.

Using the CNRM dataset, we applied three deep learning approaches to the test data after training with different amounts of data. From Figure 5, it can be observed clearly that the proposed model achieved 100% classification accuracy on 50 testing samples for each RSN class when there are more than 5 training samples for each class. Even with two samples per class, SiameseICA can still perform well in these tasks. On the other hand, inception CNN and sGAN methods were not able to achieve 100% classification accuracy even on 20 training samples. SiameseICA helped alleviate training data collection as fewer labeled examples were required to attain reasonable performance.

**FIGURE 6:**
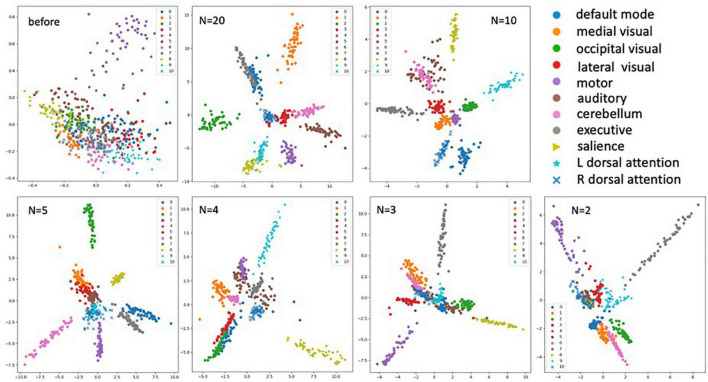
Plots of the embedding vectors projected down to 2-dimensions using PCA before and after the training with different numbers of training samples (*N* = 2, 3, 4, 5, 10, and 20). With each color representing a distinct class of the RSNs as shown by the legend.

For data visualization, all the embedded vectors were projected into a 2-dimentional space using PCA with each color representing a distinct RSN class as shown by the legend (Figure 6). We can see the embeddings of different RSN classes are mixing before training (top left plot) since the model has not learned to separate the classes out. After training with different numbers of training examples (N), we can see clear clustering of the intra-class RSNs and better separation of the inter-class RSNs. These plots indicate the model has learned to cluster the ICA images for all eleven RSNs even after reducing the dimensionality of the image features.

**FIGURE 7:**
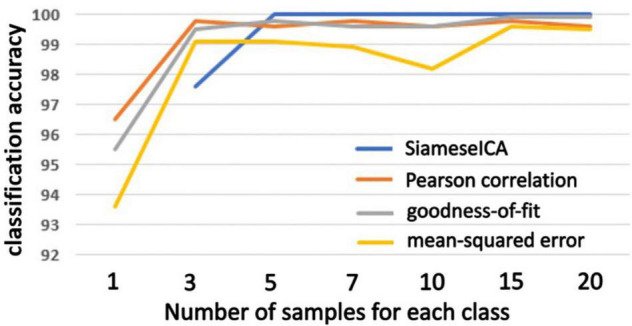
Plots of classification accuracy on the CNRM dataset with different number of examples trained for the proposed SiameseICA model and majority voting over different number of reference samples for Pearson correlation, goodness-of-fit and mean-squared error methods. Our method obtains 100% classification with five training samples.

The proposed SiameseICA method was also compared against three different template matching methods including Pearson correlation, mean squared error and goodness-of-fit by using majority voting with different numbers of reference samples for each RSN class. The SiameseICA method still used a single image as an exemplar for each class but used different numbers of training samples. As observed in Figure 7, SiameseICA outperforms the template matching methods with majority voting when trained with at least five samples for each RSN class on the CNRM dataset.

**FIGURE 8:**
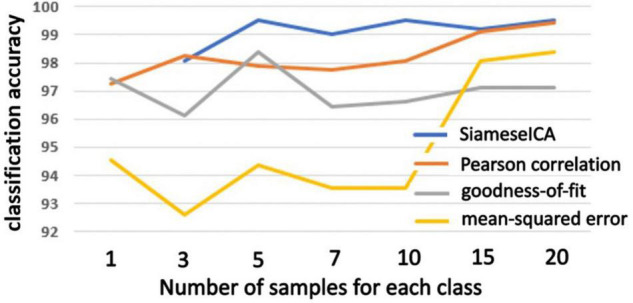
Plots of classification accuracy on the BeijingFCP dataset with different number of examples trained for the proposed SiameseICA model and majority voting over different number of reference samples for Pearson correlation, goodness-of-fit and mean-squared error methods.

The same evaluation was performed on an outside dataset (BeijingFCP) and the plots of classification accuracy are shown in Figure 8. The training samples for SiameseICA and reference samples for the template matching methods were from the CNRM dataset. Our method was as good or better than the template matching methods using majority voting when trained with more than 5 training samples.

### Generalizability, Separability, and Reproducibility

For the second phase of the evaluation, we characterized the properties of SiamaseICA aside from classification accuracy. We first examined 10 subjects from another external dataset (Milwaukee-b) acquired with a scanner and acquisition protocol different from the training data, and applied our method to extract the optimal RSNs across multiple ICA runs with varying numbers of components (20 to 99). The results for the eleven RSNs are shown in the Supplementary Material, demonstrating the feasibility of the proposed SiameseICA method for ICA classification using an input data set that is completely independent of the training data.

**FIGURE 9:**
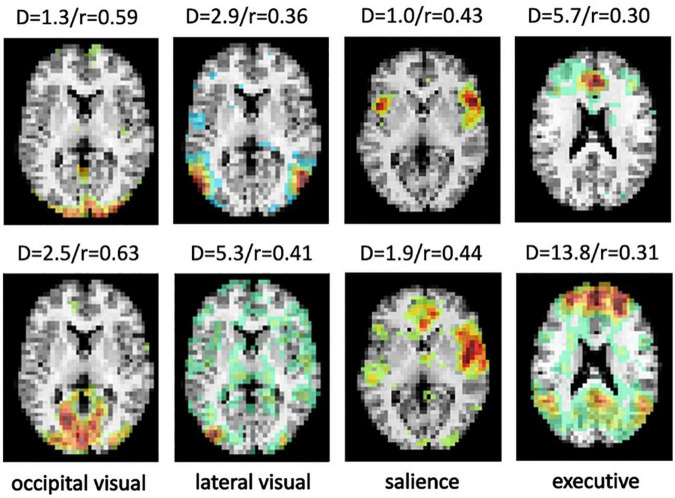
Four examples from the CNRM and Milwaukee-b datasets showing the ICA component that best represents the desired RSN labeled by SiameseICA (top row) but not correctly identified by Pearson correlation with five references per class (bottom row). Values above each image represent the metric derived by SiameseICA (left, denoted D) and Pearson correlation (right, denoted r).

A limitation of the testing set is that the selected ICA components were manually labeled with high confidence, providing a very clean data set for evaluation. However, unlike the proposed approached, template matching uses a metric that is not optimized to differentiate different networks. For example, results with noisy, split or merged ICA components can still show high Pearson Correlation. Figure 9 shows ICA components from the CNRM and Milwaukee-b datasets correctly labeled by SiameseICA but not correctly identified by Pearson Correlation.

**FIGURE 10:**
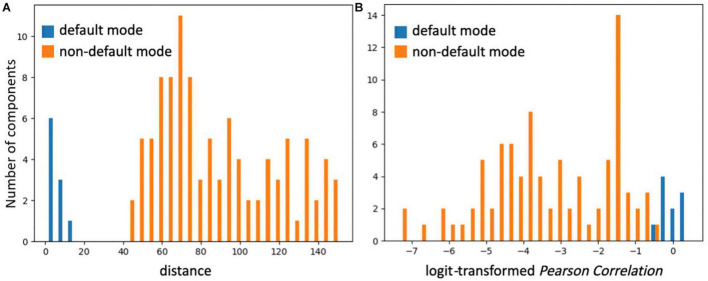
The two distributions of ICA components representing default mode and non-default mode network are visually more fully separated by **(A)** SiameseICA than **(B)** Pearson Correlation.

SiameseICA learns a representation space equipped with a metric that encourages intra-class compactness and inter-class separability. Hence, similar ICA components are assigned small distances and dissimilar components yield large distances. To examine the separability properties of the RSN classification, 10 ICA components representing the default mode network and 100 ICA components representing other, non-default mode networks from the Milwaukee-b dataset were identified by SiameseICA (trained with five samples per class) and Pearson correlation methods (with five references per class) independently. The distribution of distances for default mode components and other components using the default mode template in SiameseICA is shown in Figure 10A. Similarly, the distribution for PC is shown in Figure 10B. To expand the range of the Pearson Correlation, a logit operation was applied. As expected, the two distributions are better separated using SiameseICA than using Pearson Correlation.

**FIGURE 11:**
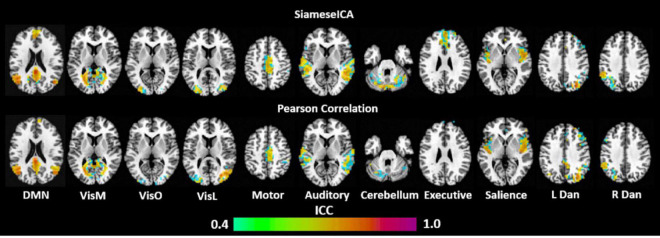
Voxel-wise intraclass correlation (ICC) values (≥0.4) showing the scan-rescan reproducibility on classifying 11 RSNs using the proposed SiameseICA model and template matching method.

Deep learning techniques are complex and can have a high range of variability, calling the reproducibility of the results into question (Renard et al., 2020). In particular, they have been shown in the literature to potentially be unstable to small variations in the input (Heaven, 2019). To quantify scan-rescan relibility of the measurements within subjects for the proposed SiameseICA model, the default mode networks of 21 subjects (each subject was scanned on two visits) from the MMRR-21 dataset were identified. A voxel-wise intraclass correlation (ICC) analysis was performed (Shrout and Fleiss, 1979) using *3dICC* (Chen et al., 2018). The interpretation of ICC values typically follows the guidelines presented in Fleiss (1986), where ICCs less than 0.4 are considered poor, ICCs between 0.4 and 0.59 are considered fair, ICCs between 0.60 and 0.74 are considered good, and ICCs greater than or equal to 0.75 are considered excellent. Results of the voxel-wise ICC analysis exceeding the pre-determined reliability threshold ICC ≥ 0.40 (McDermott et al., 2018) are shown and compared with the Pearson correlation template matching method (with five reference samples per class) (Demertzi et al., 2014) over the 11 RSNs in Figure 11. Most RSNs exhibited similar reproducibility across the two approaches. Despite being a simpler approach, Pearson correlation showed lower ICCs in some regions, such as the medial prefrontal cortex (mPFC) of the default mode network, and the frontal cortex of the executive network. Mean and standard deviation of the ICC values for 11 RSNs using the proposed SiameseICA model and Pearson correlation method were reported in Table 1. Overall, these results demonstrate that SiameseICA, despite being a deep learning approach, provides reproducibility that is at least comparable to the Pearson Correlation template-based approach (*p* = 0.57).

**TABLE 1:** Mean and standard deviation of the ICC values for 11 RSNs using the proposed SiameseICA model and Pearson Correlation (with five samples per class).

RSNs		Default mode	Medial visual	Occipital Visual	Lateral Visual	Motor	Auditory	Cerebellum	Executive	Salience	L dorsal attention	R dorsal attention	Overall average
Mean/std	SiameseICA	0.36/0.18	0.31/0.19	0.25/0.17	0.23/0.17	0.34/0.21	0.38/0.20	0.32/0.19	0.15/0.17	0.40/0.19	0.36/0.17	0.35/0.19	0.31/0.07
	Pearson correlation	0.36/0.19	0.32/0.21	0.29/0.15	0.34/0.19	0.29/0.19	0.36/0.19	0.27/0.19	0.17/0.14	0.36/0.19	0.47/0.17	0.33/0.19	0.32/0.07
													

### One-Shot Classification for the Caudate Network

**FIGURE 12:**
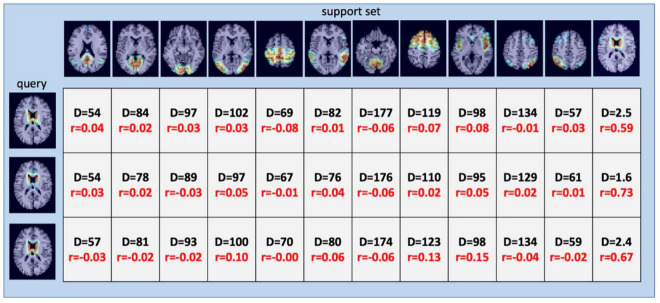
One-Shot classification of the caudate RSN using 12-way 1-shot classification. 12-way includes the 11 RSN classes of Figure 8 (DMN; MVN; OVN; LVN; AN; EN; MN, CN, SN; LDAN; and RDAN) and the additional caudate network (last column). The values represent the distance metric (color black) and Pearson correlation (color red) calculated between the support set and the queries. Distances resulting from querying the network using three examples of ICA components matching the Caudate Network (left column) are small and provide the correct classification, as does Pearson Correlation.

**FIGURE 13:**
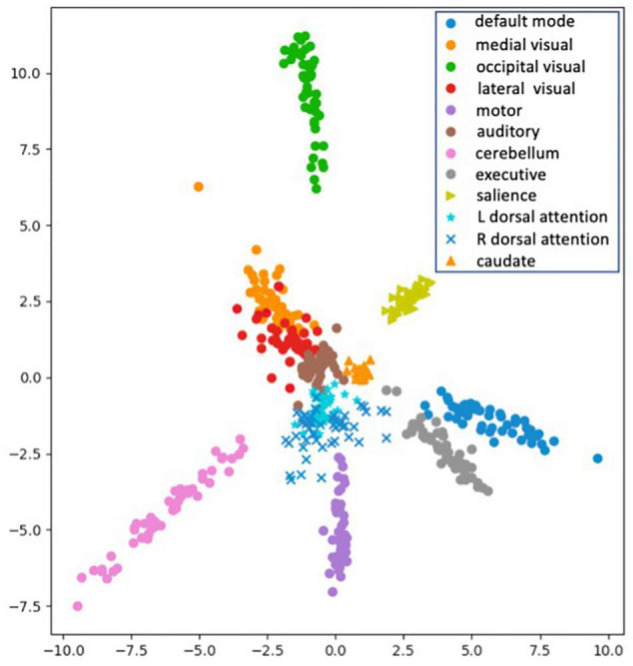
Visualization of the embedding vectors projected down to 2-dimensions using PCA for One-Shot classification.

Once SiameseICA has been trained for the classification task, the model can learn to discern a new class given only a single example without re-training the model. Experiments on one-shot classification are usually referred to as N-way 1-shot learning, where N is the number of classes (Fei-Fei et al., 2006; Vinyals et al., 2016). Given a set of support images with one image per class and a query image, the goal of one-shot classification is to be able to identify which support image the query image is most similar to. To demonstrate the discriminative potential of the learned feature mappings at one-shot classification, 21 caudate networks were manually identified from the group ICA components. One served as the reference image in the support set and the rest of the 20 images were used for the evaluation. The Siamese model was trained with 11 RSNs (without the caudate network) with five training samples. Figure 12 shows the distance metric (similarity scores) in color black and Pearson Correlation in color red calculated between the support set and the three queries for the caudate networks. Figure 13 shows the well-clustered embedding vectors of the caudate network (orange triangles) projected down to 2-dimensions using PCA in the embedding space.

### Group Comparison Between Mild TBI, Severe TBI and Healthy Subjects

As a demonstration of how this approach can be used for investigating changes in functional connectivity due to disease, we performed a small, preliminary study in data acquired from subjects with TBI. TBI can result in abnormalities of functional connectivity within key cognitive networks (Millis et al., 2001; Dikmen et al., 2009; Medaglia, 2017). We studied patients with mild TBI (49 males, 33 females, mean age: 45.3 ± 15.1 years) and severe TBI (nine males, nine females, mean age: 35.1 ± 18.0 years) compared with 35 healthy control subjects (24 males, 11 females, mean age: 41.6 ± 11.5 years) using the default mode and salience networks that were identified by the SiameseICA method with different numbers of components (*n* = 20 to 99).

**FIGURE 14:**
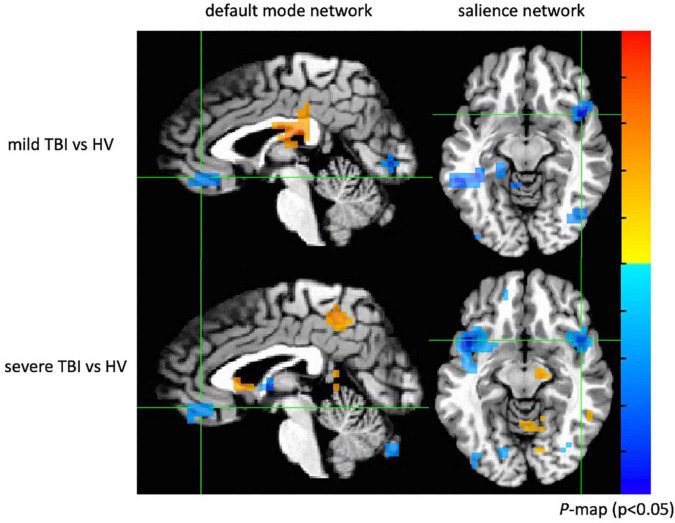
Group comparison between mild TBI, severe TBI and healthy subjects. Both mild and severe TBI groups showed significantly decreased connectivity in the ventromedial prefrontal cortex (*p* < 0.05) of the default mode network and decreased connectivity in the insular cortex (*p* < 0.05) of the salience network.

Two-sample *t* tests were implemented with age and gender as covariates to the images of the default mode and salience networks made up of ICA components from the three participant groups to generate statistical maps (Figure 14). An integrated threshold was used at a significance level of *p* < 0.05 and a cluster size of at least 15 voxels to remove false-positive error and maintain true-positive sensitivity.

When compared with the control subjects, there was significantly reduced connectivity around the ventromedial prefrontal cortex of the default mode network in patients with mild and severe TBI (*p* < 0.05; cluster size > 15) and decreased connectivity in the insular cortex of the salience network (*p* < 0.05; cluster size > 15). The results showed abnormal default mode and salience network connectivity patterns in patients with mild and severe TBI, which may provide insight into how neuronal communication and information integration are disrupted among certain key structures after brain injury. Similar findings of decreased functional connectivity within the default mode and salience network in patients with mild TBI have previously been observed (Vanhaudenhuyse et al., 2009; Mayer et al., 2011; Jackson et al., 2019; Lu et al., 2020).

## Discussion and Conclusion

In this study, we present a deep learning approach to ICA component classification based on a Siamese Network that is trained from a small dataset and still demonstrates superior performance. The architecture creates a low-dimensional embedding space for RSNs by mapping images with the same class to nearby points in a low-dimensional space using a triplet loss function. The reduced-feature representation is then used to identify images from the dataset that are most similar to a test image. We found that five examples of each RSN were sufficient to achieve accurate performance. An advantage of our approach is that it obviates the need for estimating the number of components required when applying ICA.

SiameseICA can be generalized to classify new categories without the need for additional retraining. We showed that given sufficient initial training data to define an embedding space for the support set, only one sample was required as a reference for a completely new RSN class. This approach is referred to as one-shot classification, drastically reducing the need for labeled datasets. In addition, we expect the model is more robust to class imbalance as it can be used on a dataset where very few examples exist for some classes.

The identification of the best ICA components for each RSN on different datasets showed good qualitative performance and reproducibility on independent test data, confirming the feasibility of the proposed method in research studies that may involve heterogeneous data. We also demonstrated the value of the approach in an example research study of traumatic brain injury. Using SiameseICA, we found that the default mode and salience networks were altered in mild and severe traumatic brain injury patients compared to healthy controls.

Several avenues will be explored in future work. Currently, the network outputs relative distances between the input and all of the reference images. Converting these distances into probabilities will be more intuitive as similarity scores. In addition, further optimization of the model, such as choices of hyperparameter tuning, the number of layers, number of nodes for each layer, and the learning rate of the network may yield higher performance.

## Data Availability Statement

Publicly available datasets were analyzed in this study. This data can be found here: http://fcon_1000.projects.nitrc.org/fcpClassic/FcpTable.html and http://www.nitrc.org/projects/multimodal. TBI imaging data is not yet publicly available but will be made available upon request to LC, chanle@cc.nih.gov.

## Ethics Statement

The studies involving human participants were reviewed and approved by National Institutes of Health IRB. The patients/participants provided their written informed consent to participate in this study.

## Author Contributions

YC and DP contributed to conception and design of the study and wrote sections of the manuscript. LC provided data for the study. YC labeled data for this study with guidance from CC and JB. SR helped the analysis of data. YC wrote the first draft of the manuscript. All authors contributed to manuscript revision, read, and approved the submitted version.

## Conflict of Interest

The authors declare that the research was conducted in the absence of any commercial or financial relationships that could be construed as a potential conflict of interest.

## Publisher’s Note

All claims expressed in this article are solely those of the authors and do not necessarily represent those of their affiliated organizations, or those of the publisher, the editors and the reviewers. Any product that may be evaluated in this article, or claim that may be made by its manufacturer, is not guaranteed or endorsed by the publisher.

## Footnotes

1
http://www.nitrc.org/projects/multimodal
