# Supplementary material for: Automated Classification of Resting-State fMRI ICA Components Using a Deep Siamese Network
Source: Front Neurosci. 2022 Mar 18;16:768634. doi: 10.3389/fnins.2022.768634 (PMC8971556; doi:10.3389/fnins.2022.768634)
Supplement: Supplementary file 1 [file Data_Sheet_1.docx]

**Supplementary Material:**


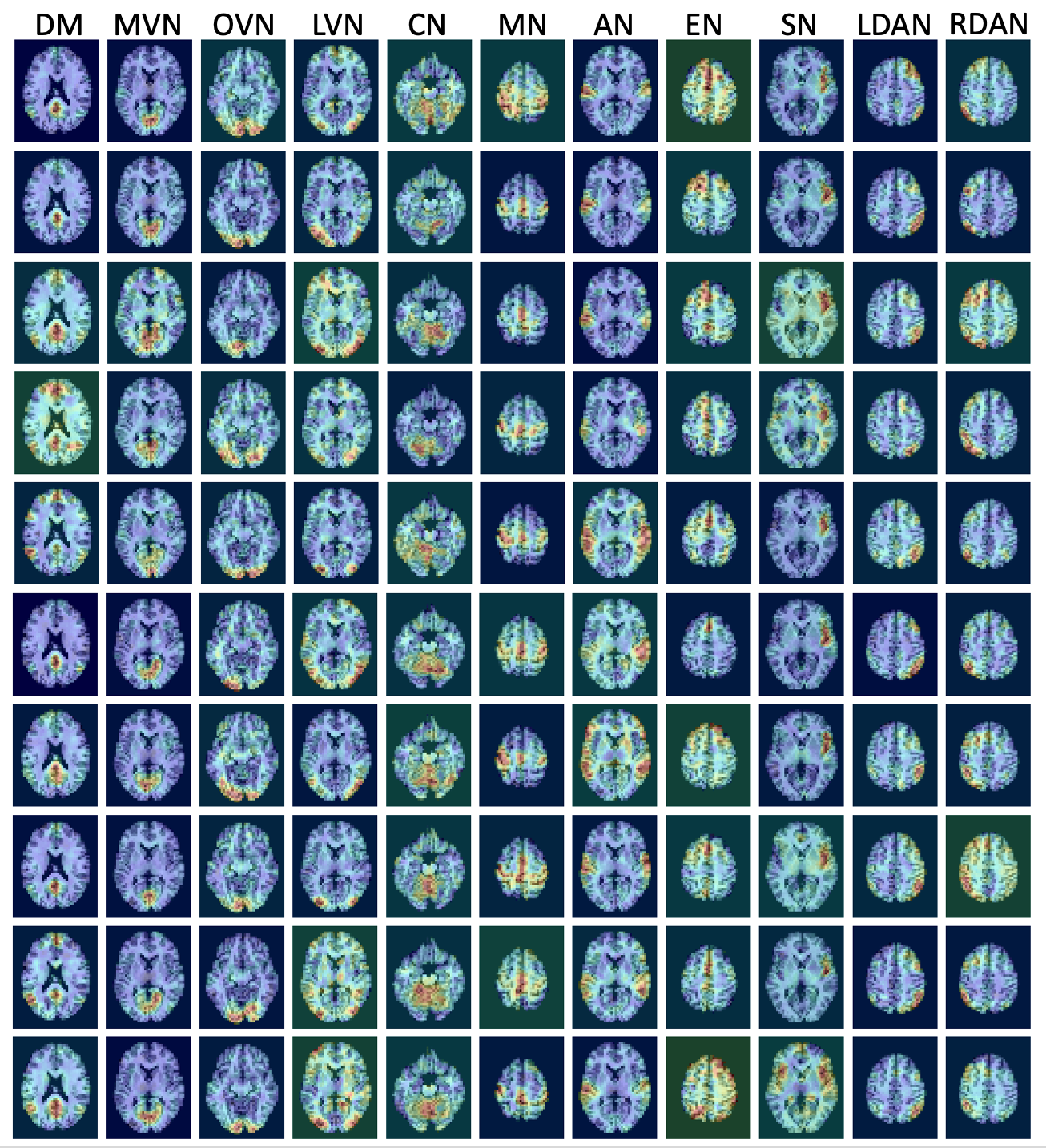


eFigure1: Examples of ICA components identified by the SiameseICA for eleven RSNs including default mode network (DMN); medial visual network (MVN); occipital visual network (OVN); lateral visual network (LVN); auditory network (AN); executive network (EN); motor network (MN), cerebellum network (CN), salience network (SN) and left dorsal attension network (LDAN) and right dorsal network (RDAN) (from left to right). Each row represents a different subject.
